# Chronotype and eating behavior in adults: associations with circadian preference across body mass index categories

**DOI:** 10.3389/fnut.2026.1816405

**Published:** 2026-05-20

**Authors:** Claudia Di Rosa, Chiara Spiezia, Alessandro Guerrini, Paolo Junior Gentili, Saverio Quarta, Silvia Manfrini, Yeganeh Manon Khazrai

**Affiliations:** 1 Research Unit of Food Science and Human Nutrition, Department of Sciences and Bio-Technologies, Università Campus Bio-Medico di Roma, Via Alvaro del Portillo, Roma, Italy; 2 IRCCS Fondazione Don Carlo Gnocchi, Florence, Italy; 3 Unit of Endocrinology and Diabetes, Fondazione Policlinico Universitario Campus Bio-Medico, Via Alvaro del Portillo, Roma, Italy

**Keywords:** appetite regulation, chronotype, circadian preference, cognitive restraint, eating behavior, emotional eating

## Abstract

Chronotype has been increasingly associated with individual differences in eating behavior and appetite regulation. While evening chronotype has often been linked to less structured eating patterns, evidence regarding specific behavioral dimensions of eating remains heterogeneous. This cross-sectional study investigated the association between chronotype and eating behavior dimensions, considering potential differences across body mass index (BMI) categories and gender. A total of 386 adults completed the Morningness–Eveningness Questionnaire (MEQ) and the Three-Factor Eating Questionnaire–Revised 18 (TFEQ-R18), assessing cognitive restraint, uncontrolled eating, and emotional eating. Participants were classified as morning, intermediate, or evening chronotypes. Morning chronotype was consistently associated with higher cognitive restraint, particularly among normal-weight individuals and women. No significant differences were observed for uncontrolled eating or emotional eating across chronotypes or BMI categories. Strong positive correlations between uncontrolled eating and emotional eating were observed across all chronotypes. Overall, chronotype appears to be primarily associated with cognitive aspects of eating regulation, highlighting links between circadian preference and day time behavioral self-regulation rather than emotional or uncontrolled eating. These findings suggest that circadian preference may contribute to distinct appetite-related behavioral profiles and support the inclusion of chronotype in behavior-oriented and personalized nutritional approaches.

## 1 Introduction

Chronotype, defined as an individual's preference for timing of sleep and activity within the 24-h day, reflects inter-individual variation in endogenous circadian rhythms. While historically studied in the context of sleep behavior, chronotype is increasingly recognized as a determinant of metabolic health and eating behavior, including the behavioral regulation of appetite, with potential implications for body weight regulation and chronic disease risk. Circadian biology research has shown that internal biological rhythms interact with feeding schedules and metabolic processes, suggesting that temporal patterns of food intake may be as relevant to health outcomes as the content and quantity of food consumed (1–3). Several studies indicate that individuals with an evening chronotype are more likely to exhibit eating behaviors associated with adverse metabolic profiles, including delayed meal timing, greater frequency of nocturnal eating, and irregular eating patterns, compared with morning chronotypes (1, 4, 5). These temporal eating patterns are often accompanied by lower diet quality, increased consumption of energy-dense foods, and higher prevalence of unhealthy lifestyle behaviors, which have been linked to higher body mass index (BMI) and increased risk of overweight and obesity (1). Mechanistic studies suggest that misalignment between endogenous circadian rhythms and behavioral cycles—such as eating late in the biological night—can disrupt peripheral clocks in metabolic tissues, leading to impaired glucose metabolism, altered lipid homeostasis, and increased adiposity (3, 6–8). As temporal food intake acts as a potent *zeitgeber* (i.e., an external environmental cue that synchronizes biological rhythms) for metabolic processes, such misalignment may contribute to long-term energy imbalance and cardiometabolic risk beyond traditional measures of diet composition (9). Beyond meal timing and metabolic outcomes, chronotype appears to influence psychological and behavioral constructs relevant to eating behavior, including appetite regulation and reward sensitivity. Although higher cognitive restraint may reflect better dietary self-regulation, restraint is not a unitary construct and may be either flexible and adaptive or rigid and potentially maladaptive, depending on the psychological context. Evidence from adult and adolescent populations suggests that later chronotypes may show higher tendencies toward dysregulated eating behaviors—such as emotional eating and uncontrolled eating—which can mediate associations between chronotype and unhealthy weight outcomes (10–12). This heterogeneity reflects differences in study design, including the widespread use of cross-sectional approaches and variability in chronotype assessment methods. Despite growing interest in chronotype as a biopsychosocial determinant of eating behavior and metabolic health, research in this area remains heterogeneous and often limited by cross-sectional design and variation in chronotype assessment. Furthermore, potential effect modification by gender and BMI category has not been consistently examined, despite evidence of gender differences in circadian regulation and eating behavior (13). Therefore, a more nuanced understanding of how chronotype relates to specific dimensions of eating behavior and appetite-related self-regulation, across diverse adult populations, is needed. Addressing these gaps has implications for personalized nutrition and chronobiologically informed interventions aimed at improving eating behavior and metabolic health.

The aim of the present study was to investigate the association between chronotype (morning, intermediate, and evening) and key dimensions of eating behavior, with a specific focus on appetite-related self-regulation, examining potential differences across BMI categories and between genders.

## 2 Materials and methods

### 2.1 Study design and participants

This exploratory observational study employed a cross-sectional design and was carried out between December 2023 and July 2025. The study was planned and reported in accordance with the STROBE guidelines for observational research. Data were obtained using an anonymous web-based survey disseminated through social media platforms (Instagram and Facebook), where the survey link was openly shared without individual invitation or tracking, allowing unrestricted access and voluntary participation. Recruitment followed a convenience sampling approach and primarily involved students enrolled at the Campus Bio-Medico University of Rome, as well as their family members. This strategy enabled the inclusion of adults across a broad age range who shared a comparable sociocultural background. Participation was voluntary and no financial or other incentives were provided. Participants were eligible if they were at least 18 years old, had a body mass index (BMI) of 18.5 kg/m^2^ or higher, provided electronic informed consent, and completed both questionnaires. Individuals were excluded if they were under 18 years of age, reported a BMI below 18.5 kg/m^2^, did not provide consent, or submitted incomplete responses. Individuals with a body mass index (BMI) below 18.5 kg/m^2^ were excluded to reduce heterogeneity and potential confounding effects. Underweight status is frequently associated with distinct eating behavior profiles, including elevated dietary restraint and disordered eating tendencies, which could have biased the assessment of appetite-related behaviors in a non-clinical adult sample.

The survey included two validated instruments administered online: the Three-Factor Eating Questionnaire–Revised 18 (TFEQ-R18) (14) used to evaluate eating behavior, and the Morningness–Eveningness Questionnaire (MEQ) (15), used to assess chronotype. Participants also provided self-reported demographic and anthropometric information, including age, gender, height, weight, and the presence of medical conditions. Due to the open dissemination of the survey through institutional and informal networks, an exact response rate could not be determined. Several measures were adopted to enhance data quality, including anonymous participation with single submission per device, mandatory informed consent, and inclusion of only fully completed questionnaires. Responses were checked for plausibility and internal consistency, and no duplicate or clearly inconsistent entries were identified. As with all voluntary online surveys, self-selection bias cannot be excluded, and the convenience sampling strategy may limit the generalizability of the findings.

The study size was determined by the number of participants who responded to the online survey during the data collection period.

### 2.2 Ethics

The study was approved by the Ethics Committee of the Campus Bio-Medico University of Rome (protocol 61.23 OSS, 17/05/2023). All participants provided electronic informed consent before accessing the survey.

### 2.3 Questionnaires

#### 2.3.1 Three-factor eating questionnaire–revised 18 (TFEQ-R18)

The Three-Factor Eating Questionnaire–Revised 18 (TFEQ-R18) (14) is a shortened and validated version of the original 51-item questionnaire developed by Stunkard and Messick (15). The TFEQ-R18 assesses three core dimensions of eating behavior: Cognitive Restraint (CR, 6 items), Uncontrolled Eating (UE, 9 items), and Emotional Eating (EE, 3 items). Cognitive restraint refers to the conscious restriction of food intake to control body weight; uncontrolled eating describes a tendency to overeat due to loss of control over intake; emotional eating refers to eating in response to negative emotions rather than physiological hunger. The questionnaire consists of 18 items rated on a 4-point Likert scale. Due to its brevity, reliability, and ease of administration, the TFEQ-R18 is widely used in both clinical practice and research settings to evaluate eating behavior patterns.

#### 2.3.2 Morningness–eveningness questionnaire (MEQ)

Chronotype was assessed using the Morningness–Eveningness Questionnaire (MEQ), developed by Horne and Östberg (16). The MEQ consists of 19 multiple-choice items focusing on daily preferences and habitual timing of sleep–wake behavior and activities. Based on the total score, participants were classified into three chronotype categories: evening type (scores 16–41), intermediate type (scores 42–58), and morning type (scores 59–86). These cut-offs are based on the original classification by Horne and Östberg and are widely used in chronotype research.

### 2.4 Statistical analysis

Statistical analyses were performed using GraphPad version 10.4.0. Continuous variables were expressed as mean ± standard deviation (SD). Prior to parametric analyses, data distribution was assessed using visual inspection of histograms and Q–Q plots, and homogeneity of variances was evaluated using Levene's test. Given the exploratory nature of the study and the robustness of ANOVA to moderate deviations from normality, parametric tests were retained. However, results should be interpreted with caution, particularly in smaller subgroups.

Differences in eating behavior scores among chronotype groups were evaluated using one-way analysis of variance (ANOVA), followed by Tukey's *post-hoc* test for multiple comparisons. Given the multiple comparisons performed across chronotype groups and stratified analyses, no formal adjustment for multiple testing was applied; therefore, findings should be interpreted as exploratory and hypothesis-generating rather than confirmatory. Analyses were conducted both in the overall sample and after stratification by BMI and gender.

The relationship between eating behavior dimensions and chronotype were assessed using Spearman's rank correlation coefficient. A two-tailed *p* value < 0.05 was considered statistically significant. No a priori sample size or statistical power calculation was performed due to the exploratory nature of the study. No formal assessment of missing data patterns was performed, as only fully completed questionnaires were included in the analyses.

## 3 Results

**Figure 1:**
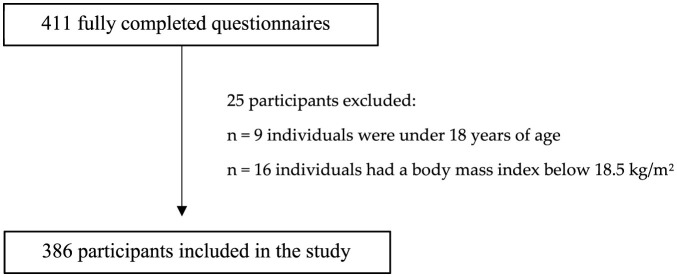
Flow chart of participants. The total number of submitted questionnaires could not be determined due to the open dissemination of the survey via social media.

A total of 411 fully completed questionnaires were available for screening. Of these, 25 were excluded because participants were under 18 years of age (*n* = 9) or had a body mass index (BMI) below 18.5 kg/m^2^ (*n* = 16). The final study sample therefore consisted of 386 participants, including 279 women (72%) and 107 men (28%) (Figure 1).

The mean age of the participants was 31.4 ± 12.3 years, the mean height of the participants included in the study was 1.7 ± 0.1 m, the mean body weight was 68.2 ± 12.8 kg, and the mean BMI was 23.5 ± 3.3 kg/m^2^.

Of the 386 participants, 305 (79%) reported no medical conditions. The remaining 81 participants (21%) reported at least one medical condition. Among these, the most frequently reported conditions were dyslipidemia (21%), hypertension (18%), irritable bowel syndrome (15%), thyroid disorders (9%), celiac disease (4%), type 1 diabetes mellitus (3%), and type 2 diabetes mellitus (2%). The remaining 27% reported other conditions, epilepsy, polycystic ovary syndrome (PCOS), gastroesophageal reflux disease (GERD), Raynaud's syndrome, and other less prevalent disorders.

Analyses were primarily conducted according to chronotype, with additional exploratory stratifications by BMI and gender.

### 3.1 Stratification by chronotype

Based on MEQ scores, the 386 participants were classified into three chronotype groups. The evening chronotype group included 64 participants (17%), of whom 36 were women (56%) and 28 were men (44%). The intermediate chronotype group comprised 224 participants (58%), including 147 women (66%) and 77 men (34%). The morning chronotype group consisted of 98 participants (25%), with 62 women (63%) and 36 men (37%).

### 3.2 Stratification by BMI

The study population was stratified according to body mass index (BMI) into normal-weight (BMI 18.5–24.9 kg/m^2^) and overweight (BMI ≥ 25.0 kg/m^2^) groups. Among evening chronotypes, 46 participants (72%) were classified as normal weight and 18 (28%) as overweight. In the intermediate chronotype group, 166 participants (74%) were normal weight and 58 (26%) were overweight. Among morning chronotypes, 67 participants (68%) were normal weight, while 31 (32%) were overweight.

### 3.3 Stratification by gender

When stratified by gender and body mass index (BMI), distinct distributions were observed within each chronotype group. Among evening chronotypes, 28 participants were normal-weight women (44%), 8 were overweight women (12%), 18 were normal-weight men (28%), and 10 were overweight men (16%). Within the intermediate chronotype group, 115 participants were normal-weight women (51%), 32 were overweight women (14%), 51 were normal-weight men (23%), and 26 were overweight men (12%). Among morning chronotypes, 45 participants were normal-weight women (46%), 17 were overweight women (17%), 22 were normal-weight men (23%), and 14 were overweight men (14%).

### 3.4 Comparison of eating behavior among the three chronotypes stratified by BMI

**Figure 2:**
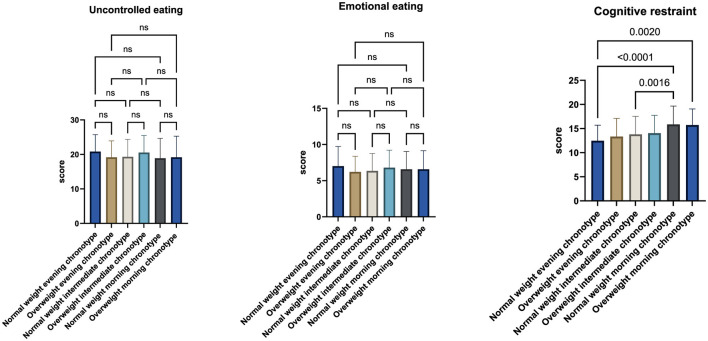
Mean scores of uncontrolled eating, emotional eating, and cognitive restraint across chronotype groups, stratified by body mass index (BMI) categories (normal weight and overweight). Analyses were performed using one-way analysis of variance (ANOVA) followed by Tukey's *post-hoc* test for multiple comparisons.

When comparing TFEQ-R18 subscale scores across chronotype groups, no statistically significant differences were observed for uncontrolled eating or emotional eating. In contrast, significant differences emerged for cognitive restraint. Specifically, cognitive restraint scores were higher in overweight morning chronotypes compared with normal-weight evening chronotypes (*p* = 0.0020), in normal-weight morning chronotypes compared with normal-weight intermediate chronotypes (*p* = 0.0016), and in normal-weight morning chronotypes compared with normal-weight evening chronotypes (*p* < 0.0001) (Figure 2).

### 3.5 Comparison between men and women within the same chronotype divided by BMI

When comparing women and men within evening, intermediate, and morning chronotype groups, stratified by BMI, no statistically significant differences were observed across the three TFEQ-R18 subscales. Within the evening chronotype group, overweight women showed higher mean cognitive restraint scores (14.9 ± 3.9) compared with the other subgroups, whose scores did not exceed 12.7. In addition, normal-weight women with an evening chronotype exhibited the highest mean scores for uncontrolled eating (21.4 ± 5.3) and emotional eating (7.6 ± 2.7) within this chronotype. These subgroup-specific descriptive patterns, particularly in overweight evening men, should be interpreted with caution given the very small subgroup size (*n* = 10), which limits the reliability of statistical comparisons and precludes robust conclusions. Within the intermediate chronotype group, no statistically significant differences were observed across the three TFEQ-R18 subscales when stratified by gender and BMI. However, overweight men exhibited the highest mean scores for cognitive restraint, uncontrolled eating, and emotional eating within this chronotype group. Within the morning chronotype group, variability in TFEQ-R18 subscale scores was observed across gender and BMI categories; however, no statistically significant differences were detected.

### 3.6 Eating behavior in men according to chronotype and body mass index

**Figure 3:**
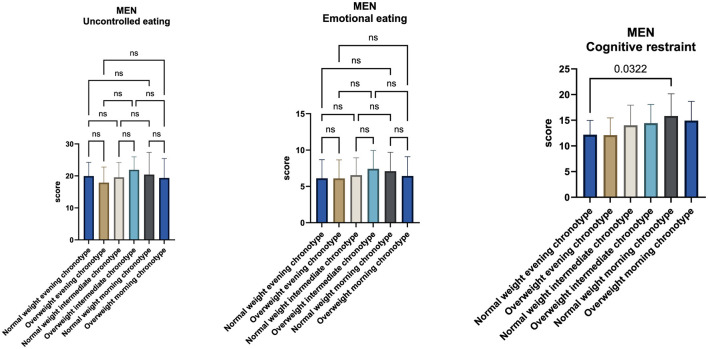
Comparison of uncontrolled eating, emotional eating, and cognitive restraint scores across chronotype groups in the male sample, stratified by body mass index (BMI). Analyses were performed using one-way analysis of variance (ANOVA) followed by Tukey's *post-hoc* test for multiple comparisons.

Subsequent analyses focused on gender-specific differences. In the male sample, no statistically significant differences were observed in uncontrolled eating or emotional eating scores across chronotype groups or BMI categories (Figure 3).

Within the cognitive restraint subscale, a statistically significant difference was observed between normal-weight evening and normal-weight morning chronotypes (*p* = 0.0322). Normal-weight morning chronotypes exhibited higher cognitive restraint scores (15.8 ± 4.3) compared with normal-weight evening chronotypes (12.2 ± 2.8).

### 3.7 Eating behavior in women according to chronotype and body mass index

**Figure 4:**
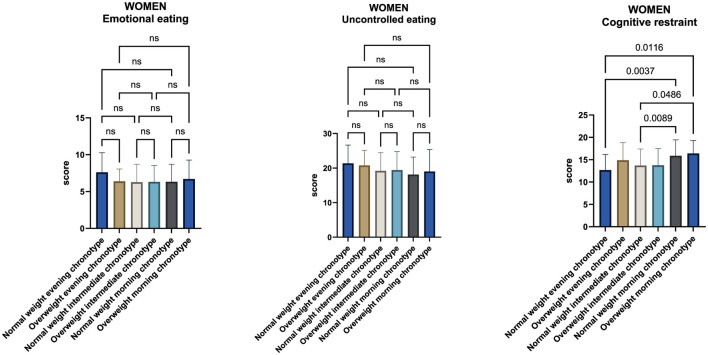
Comparison of uncontrolled eating, emotional eating, and cognitive restraint scores across chronotype groups in the female sample. Analyses were performed using one-way analysis of variance (ANOVA) followed by Tukey's *post-hoc* test for multiple comparisons.

As observed in the male sample, no statistically significant differences were detected among women across chronotype groups with respect to uncontrolled eating or emotional eating. In contrast, several statistically significant differences emerged in cognitive restraint scores (Figure 4).

Normal-weight women with a morning chronotype exhibited higher cognitive restraint scores (15.9 ± 3.6) compared with both normal-weight evening women (12.7 ± 3.5) and normal-weight intermediate women (13.1 ± 3.7). In addition, overweight morning women showed higher cognitive restraint scores (16.4 ± 2.9) than both normal-weight intermediate women (13.1 ± 3.7) and normal-weight evening women (12.2 ± 2.8).

### 3.8 Correlations

To further examine the relationships among eating behavior dimensions within each chronotype, correlation analyses were performed. Within the evening chronotype group, a strong and statistically significant positive correlation was observed between uncontrolled eating and emotional eating (*r* = 0.610, *p* < 0.0001). No significant correlations were detected between eating behavior dimensions when stratified by BMI.

Several significant correlations were identified within the intermediate chronotype group. Consistent with observations in the evening chronotype, a strong positive correlation was found between uncontrolled eating and emotional eating (*r* = 0.619, *p* < 0.0001). Additionally, a moderate positive correlation was observed between cognitive restraint and emotional eating (*r* = 0.300, *p* < 0.0001), while a weak but statistically significant correlation emerged between cognitive restraint and uncontrolled eating (*r* = 0.168, *p* = 0.012).

Within the morning chronotype group, two statistically significant positive correlations were identified. A strong positive correlation was observed between uncontrolled eating and emotional eating (*r* = 0.735, *p* < 0.0001). In addition, a moderate positive correlation was found between cognitive restraint and emotional eating (*r* = 0.354, *p* < 0.0001) (Table 1).

**Table 1:** Spearman correlations between eating behavior dimensions within chronotype groups.

Variables	Correlation coefficient (r)	*p*-value	Interpretation
EVENING CHRONOTYPES			
Uncontrolled eating vs. emotional eating	0.620	< 0.0001	Strong positive correlation
INTERMEDIATE CHRONOTYPES			
Uncontrolled eating vs. emotional eating	0.619	< 0.0001	Strong positive correlation
Cognitive restraint vs. emotional eating	0.300	0.0001	Moderate positive correlation
Cognitive restraint vs. uncontrolled eating	0.168	0.012	Weak positive correlation
MORNING CHRONOTYPES			
Uncontrolled eating vs. emotional eating	0.735	< 0.0001	Strong positive correlation
Cognitive restraint vs. emotional eating	0.354	< 0.0001	Moderate positive correlation

## 4 Discussion

Previous studies conducted on large populations indicate that chronotype plays a relevant role in shaping eating behavior and dietary habits. Individuals with an evening chronotype generally report later bedtimes, greater variability in breakfast timing, higher consumption of caffeinated and alcoholic beverages, and overall poorer diet quality compared with morning chronotypes (1, 2, 17). In contrast, morning chronotypes tend to follow more regular and nutritionally balanced eating patterns, with higher intakes of proteins, carbohydrates, vitamins, and minerals (1, 2, 18, 19).

The findings of the present study partially align with this literature. While no statistically significant differences were observed across chronotypes for uncontrolled eating and emotional eating, individuals with an evening chronotype tended to exhibit higher uncontrolled eating scores, consistent with previous observations of less regulated eating patterns among late chronotypes (1, 2, 18, 19). However, the most distinctive result concerned cognitive restraint, which was significantly higher in morning chronotypes, particularly among normal-weight individuals and women.

This finding is consistent with evidence suggesting that morning chronotypes are more likely to adopt structured and rule-based eating behaviors (4, 18). At the same time, it expands existing knowledge by highlighting that higher cognitive restraint is not necessarily associated with healthier outcomes, as rigid dietary control may coexist with emotional vulnerability and risk of disinhibited eating (20, 21). From a clinical nutrition perspective, higher cognitive restraint should not automatically be interpreted as beneficial. Flexible restraint may support meal planning and self-regulation, whereas rigid restraint may increase stress around eating and may coexist with emotional vulnerability or later disinhibition. Since the TFEQ-R18 does not distinguish between flexible and rigid restraint, the present findings should be interpreted cautiously. This distinction is particularly relevant in clinical practice, where understanding whether restraint reflects adaptive regulation or maladaptive rigidity is essential for nutritional counseling. Notably, direct comparisons between chronotype and cognitive restraint are still limited in the literature, and few studies have examined this relationship while simultaneously accounting for BMI and gender. In this respect, the present study contributes novel evidence to an underexplored area.

The association between cognitive restraint and BMI observed in this study is consistent with prior research indicating that dietary restraint often increases with higher body weight (22). Longitudinal studies further suggest a bidirectional relationship, whereby greater adiposity predicts subsequent increases in cognitive restraint rather than restraint leading to weight loss (23, 24). This mechanism may explain the elevated restraint scores observed among overweight participants, particularly among morning chronotypes.

In contrast, emotional eating was not associated with higher BMI in the present sample, as higher scores were observed among normal-weight individuals rather than overweight participants. This pattern may suggest that cognitive restraint in morning chronotypes is more strongly related to behavioral traits associated with circadian preference than to body weight status per se. This finding differs from some previous reports showing a positive association between emotional eating and BMI (22), but aligns with studies suggesting that emotional eating may not consistently translate into higher body weight, especially in younger or non-clinical populations (21). Differences in age, sample composition, and study design may account for these discrepancies.

Gender-related patterns observed in the present study should be interpreted with caution. Although no statistically significant differences emerged between men and women across BMI categories, women—particularly those with morning and evening chronotypes—tended to report higher mean scores for cognitive restraint and emotional eating. This descriptive pattern is consistent with previous evidence indicating that women generally report higher levels of dietary restraint and emotional eating than men (25, 26). Together, these observations support the potential role of sociocultural and psychological factors in shaping eating behavior among women, including greater dietary monitoring and sensitivity to body image norms (27–31). Correlation analyses further reinforced the complex interplay between eating behavior dimensions. Strong associations between uncontrolled eating and emotional eating were observed across all chronotypes, consistent with previous studies suggesting shared underlying mechanisms (26, 32). Additional correlations between cognitive restraint and emotional eating in morning and intermediate chronotypes highlight the coexistence of dietary control and emotional vulnerability, supporting models that conceptualize eating behavior as a dynamic balance rather than independent traits (20, 21, 33).

Overall, this study suggests that cognitive restraint is the eating behavior dimension most sensitive to chronotype, gender, and BMI, whereas uncontrolled eating and emotional eating appear more stable across circadian preferences. Morning chronotype, in particular, is associated with greater dietary control, which may represent either an adaptive or potentially maladaptive strategy depending on context. These results should be interpreted with caution, particularly in smaller subgroups, as limited sample sizes may reduce statistical power and increase the risk of spurious or unstable findings (34).

Taken together, the present findings underline the importance of considering chronotype in the study of eating behavior and support the integration of circadian preferences into personalized nutritional and preventive strategies.

## 5 Limitations

First, the exploratory cross-sectional design of this study does not allow any inference of causality or temporal direction between chronotype and eating behavior. Consequently, the observed associations should be interpreted as descriptive and non-causal, and not as evidence of cause–effect relationships or clinical implications related to obesity.

Second, the study relied exclusively on self-reported data, including anthropometric measures and questionnaire-based assessments. Because height and weight were self-reported, BMI estimates may have been affected by reporting bias, including possible under-reporting of body weight and over-reporting of height. Although validated instruments were used to evaluate chronotype and eating behavior, self-reporting may be affected by recall bias or social desirability and may not fully reflect actual behaviors or physiological characteristics.

Third, participants were recruited through online platforms and informal social networks and were predominantly university students and their family members. This convenience sampling strategy may have resulted in a sample with relatively homogeneous sociodemographic characteristics, thereby limiting the generalizability of the findings to the broader adult population. In addition, voluntary participation may have introduced self-selection bias. Moreover, body mass index (BMI) was used as the sole anthropometric indicator. While this choice is consistent with the online observational design of the study, BMI does not allow differentiation between fat mass and lean mass. Future studies conducted in in-person settings and including direct assessments of body composition (e.g., bioelectrical impedance analysis) could provide a more nuanced interpretation of eating behavior patterns in relation to chronotype.

Finally, although analyses accounted for BMI and gender, other potentially relevant factors—such as age, socioeconomic status, educational level, physical activity, sleep characteristics, and lifestyle habits—were not included and may have acted as residual confounders. Sleep duration was not assessed in the present survey and therefore could not be included among the study variables, although it may influence both chronotype-related behaviors and appetite regulation. No a priori sample size or power calculation was performed, and some subgroup analyses may have been underpowered, with small subgroup sample sizes potentially resulting in spurious statistically significant findings. Thus, findings from this study should be interpreted with caution.

## 6 Conclusions

Chronotype appears to be associated mainly with cognitive restraint, with morning chronotypes showing higher levels of dietary control, particularly among women and normal-weight individuals, while uncontrolled and emotional eating remained stable across groups.

The strong association between uncontrolled and emotional eating highlights the interconnected nature of appetite-related behaviors, and the coexistence of restraint and emotional eating suggests that higher dietary control may not always reflect adaptive regulation.

Overall, these findings support the role of chronotype as a behavioral factor in appetite regulation and suggest its potential relevance for personalized, behavior-oriented nutritional strategies. However, these implications should be interpreted cautiously, as chronotype-based approaches are not currently included in nutritional guidelines.

## Data Availability

The datasets generated and analyzed during the current study are not publicly available due to privacy and data protection regulations, as the data contain information that could potentially allow participant identification. The data are available from the corresponding author upon reasonable request and in accordance with institutional and ethical approval. Requests to access these datasets should be directed to c.dirosa@unicampus.it.

## References

[1] Teixeira GP Guimarães KC Soares AGN Marqueze EC Moreno CRC Mota MC . Role of chronotype in dietary intake, meal timing, and obesity: a systematic review. Nutr Rev. (2023) 81:75–90. doi: 10.1093/nutrit/nuac044 35771674

[2] van der Merwe C Münch M Kruger R. Chronotype differences in body composition, dietary intake and eating behavior outcomes: a scoping review. Adv Nutr. (2022) 13:2357–405. doi: 10.1093/advances/nmac093 36041181 PMC9776742

[3] Xiao Q Garaulet M Scheer FAJ. Meal timing and obesity: interactions with macronutrient intake and chronotype. Int J Obes. (2019) 43:1730–41. doi: 10.1038/s41366-018-0284-x PMC6669101 30705391

[4] Maukonen M Kanerva N Partonen T Kronholm E Tapanainen H Kontto J . Chronotype differences in timing of energy and macronutrient intakes: a population-based study. Obesity (Silver Spring). (2016) 33:771–80. doi: 10.1002/oby.21747 28229553

[5] Arora T Taheri S. Associations among late chronotype, body mass index and dietary behaviors in adolescents and adults: a systematic review and meta-analysis. Sleep Med Rev. (2015) 39:39–44. doi: 10.1038/ijo.2014.157 25135376

[6] Wehrens SMT Christou S Isherwood C Middleton B Gibbs MA Archer SN . Meal timing regulates the human circadian system. Curr Biol. (2017) 27:1768–75. doi: 10.1016/j.cub.2017.04.059 28578930 PMC5483233

[7] Morris CJ Yang JN Garcia JI Myers S Bozzi I Wang W . Endogenous circadian system and circadian misalignment impact glucose tolerance via separate mechanisms in humans. Proc Natl Acad Sci U S A. (2015) 112:E2225–34. doi: 10.1073/pnas.1418955112 25870289 PMC4418873

[8] Wittmann M Dinich J Merrow M Roenneberg T. Social jetlag: misalignment of biological and social time. Chronobiol Int. (2006) 23:497–509. doi: 10.1080/07420520500545979 16687322

[9] Longo VD Panda S. Fasting, circadian rhythms, and time-restricted feeding in healthy lifespan. Cell Metab. (2016) 23:1048–59. doi: 10.1016/j.cmet.2016.06.001 27304506 PMC5388543

[10] Maukonen M Kanerva N Partonen T Männistö S. Chronotype and energy intake timing in relation to changes in anthropometrics: a 7-year follow-up study in adults. Chronobiol Int. (2019) 36:27–41. doi: 10.1080/07420528.2018.1515772 30212231

[11] Baron KG Reid KJ. Circadian misalignment and health. Int Rev Psychiatry. (2014) 26:139–54. doi: 10.3109/09540261.2014.911149 24892891 PMC4677771

[12] Fabbian F Zucchi B De Giorgi A Tiseo R Boari B Salmi R . Chronotype, gender and general health. Chronobiol Int. (2016) 33:863–82. doi: 10.1080/07420528.2016.1176927 27148626

[13] Lok R Qian J Chellappa SL. Sex differences in sleep, circadian rhythms, and metabolism: implications for precision medicine. Sleep Med Rev. (2024) 75:101926. doi: 10.1016/j.smrv.2024.101926 38564856

[14] Rossi AA Pietrabissa G Castelnuovo G Mannarini S. Cognitive restraint, uncontrolled eating, and emotional eating. The Italian version of the three factor eating questionnaire-revised 18 (TFEQ-R-18): a three-step validation study. Eat Weight Disord. (2024) 29:16. doi: 10.1007/s40519-024-01642-y 38402372 PMC10894126

[15] Stunkard AJ Messick S. The three-factor eating questionnaire to measure dietary restraint, disinhibition and hunger. J Psychosom Res. (1985) 29:71–83. doi: 10.1016/0022-3999(85)90010-8 3981480

[16] Horne JA Ostberg O. A self-assessment questionnaire to determine morningness-eveningness in human circadian rhythms. Int J Chronobiol. (1976) 4:97–110. doi: 10.1037/t02254-000 1027738

[17] Fleig D Randler C. Association between chronotype and diet in adolescents based on food logs. Eat Behav. (2009) 10:115–8. doi: 10.1016/j.eatbeh.2009.03.002 19447353

[18] Sato-Mito N Sasaki S Murakami K Okubo H Takahashi Y Shibata S . Freshmen in dietetic courses study II group. The midpoint of sleep is associated with dietary intake and dietary behavior among young Japanese women. Sleep Med. (2011) 12:289–94. doi: 10.1016/j.sleep.2010.09.012 21296614

[19] Sato-Mito N Shibata S Sasaki S Sato K. Dietary intake is associated with human chronotype as assessed by both morningness-eveningness score and preferred midpoint of sleep in young Japanese women. Int J Food Sci Nutr. (2011) 62:525–32. doi: 10.3109/09637486.2011.560563 21495902

[20] Westenhoefer J. Dietary restraint and disinhibition: is restraint a homogeneous construct? Appetite. (1991) 16:45–55. doi: 10.1016/0195-6663(91)90110-E 2018403

[21] Dakanalis A Mentzelou M Papadopoulou SK Papandreou D Spanoudaki M Vasios GK . The association of emotional eating with overweight/obesity, depression, anxiety/stress, and dietary patterns: a review of the current clinical evidence. Nutrients. (2023) 15:1173. doi: 10.3390/nu15051173 36904172 PMC10005347

[22] Anglé S Engblom J Eriksson T Kautiainen S Saha M-T Lindfors P . Three factor eating questionnaire-R18 as a measure of cognitive restraint, uncontrolled eating and emotional eating in a sample of young Finnish females. Int J Behav Nutr Phys Act. (2009) 6:41. doi: 10.1186/1479-5868-6-41 19615047 PMC2720907

[23] Snoek HM van Strien T Janssens JM Engels RC. Emotional, external and restrained eating behaviour and BMI trajectories in adolescence. Appetite. (2008) 51:717–22. doi: 10.1016/j.appet.2013.03.014 23571047

[24] Chavance M Escolano S Romon M Basdevant A de Lauzon-Guillain B Charles M . Latent variables and structural equation models for longitudinal relationships: an illustration in nutritional epidemiology. BMC Med Res Methodol. (2010) 10:37. doi: 10.1186/1471-2288-10-37 20433707 PMC2873513

[25] Elfhag K Linné Y. Gender differences in associations of eating pathology between mothers and their adolescent offspring. Obes Res. (2005) 13:1070–6. doi: 10.1038/oby.2005.125 15976150

[26] Keskitalo K Tuorila H Spector TD Cherkas LF Knaapila A Kaprio J . The three-factor eating questionnaire, body mass index, and responses to sweet and salty fatty foods: a twin study of genetic and environmental associations. Am J Clin Nutr. (2008) 88:263–71. doi: 10.1093/ajcn/88.2.263 18689360

[27] Malloy JA Kazenbroot-Phillips H Roy R. Associations between body image, eating behaviors, and diet quality among young women in New Zealand: the role of social media. Nutrients. (2024) 16:3517. doi: 10.3390/nu16203517 39458512 PMC11510262

[28] Spinelli S Dinnella C Tesini F Bendini A Braghieri A Proserpio C . Gender differences in fat-rich meat choice: influence of personality and attitudes. Nutrients. (2020) 12:1374. doi: 10.3390/nu12051374 32403419 PMC7285107

[29] Feraco A Armani A Amoah I Guseva E Camajani E Gorini S . Assessing gender differences in food preferences and physical activity: a population-based survey. Front Nutrition. (2024) 11:1348456. doi: 10.3389/fnut.2024.1348456 PMC10912473 38445208

[30] Lombardo M Aulisa G Padua E Annino G Iellamo F Pratesi A . Gender differences in taste and foods habits. Nutrition Food Sci. (2020) 50:229–39. doi: 10.1108/NFS-04-2019-0132

[31] Baker AH Wardle J. Sex differences in fruit and vegetable intake in older adults. Appetite. (2003) 40:269–75. doi: 10.1016/S0195-6663(03)00014-X 12798784

[32] Canetti L Bachar E Berry EM. Food and emotion. Behav Processes. (2002) 60:157–64. doi: 10.1016/S0376-6357(02)00082-7 12426067

[33] De Lauzon B Romon M Deschamps V Lafay L Borys JM Karlsson J . The three-factor eating questionnaire-R18 is able to distinguish among different eating patterns in a general population. J Nutr. (2004) 134:2372–80. doi: 10.1093/jn/134.9.2372 15333731

[34] Cao Y Chen RC Katz AJ. Why is a small sample size not enough? Oncologist. (2024) 29:761–3. doi: 10.1093/oncolo/oyae162 38934301 PMC11379640

